# Increased Soluble VCAM-1 and Normal P-Selectin in Cystic Fibrosis: a Cross-Sectional Study

**DOI:** 10.1007/s00408-017-0029-y

**Published:** 2017-06-23

**Authors:** Jan K. Nowak, Irena Wojsyk-Banaszak, Edyta Mądry, Andrzej Wykrętowicz, Patrycja Krzyżanowska, Sławomira Drzymała-Czyż, Agata Nowicka, Andrzej Pogorzelski, Ewa Sapiejka, Wojciech Skorupa, Mariusz Szczepanik, Aleksandra Lisowska, Jaroslaw Walkowiak

**Affiliations:** 1 Department of Pediatric Gastroenterology and Metabolic Diseases, Poznan University of Medical Sciences, Poznan, Poland; 2 Department of Pediatric Pneumonology, Allergology and Clinical Immunology, Poznan University of Medical Sciences, Poznan, Poland; 3 Department of Physiology, Poznan University of Medical Sciences, Poznan, Poland; 4 Department of Cardiology-Intensive Therapy, Poznan University of Medical Sciences, Poznan, Poland; 5 Department of Pulmonology, Allergology and Respiratory Oncology, Poznan University of Medical Sciences, Poznan, Poland; 6 Department of Pneumology and Cystic Fibrosis, Institute of Tuberculosis and Lung Diseases, Rabka, Poland; 7 Outpatient Clinic for CF Patients, Gdansk, Poland; 8 Department of Lung Diseases, National Institute for Tuberculosis and Lung Diseases, Warsaw, Poland

**Keywords:** Atherosclerosis, Atherogenesis, CD62, CD106, GMP140, LECAM3, Pancreatic insufficiency

## Abstract

**Purpose:**

As life expectancy in cystic fibrosis (CF) increases, questions regarding its potential impact on cardiovascular health arise. Soluble vascular cell adhesion molecule 1 (sVCAM-1), P-selectin (sP-selectin) are proposed as biomarkers of cardiovascular disease. We aimed to: compare their concentrations in clinically stable CF patients and healthy subjects (HS) and verify whether they independently correlate with CF characteristics.

**Methods:**

Serum sVCAM-1 and sP-selectin levels were measured using ELISA. CF was characterized using: forced expiratory volume in 1 s, exocrine pancreatic and CF-related liver disease status, *Pseudomonas aeruginosa* colonization, serum high-sensitivity C-reactive protein, and body mass index (BMI). *CFTR* genotypes were classified as severe (classes I and II) or other.

**Results:**

108 CF patients and 51 healthy subjects volunteered for the study. In the CF group BMI was lower (median [IQR]: 20.5 kg/m^2^ [18.4–22.2] vs. 21.6 kg/m^2^ [19.9–23.4], *p* = 0.02) and hsCRP levels were higher (3.6 mg/L [1.1–7.1] vs. 0.5 mg/dL [0.3–1.0], *p* < 10^−10^). While sVCAM-1 concentrations were greater in CF patients (1018 ng/mL [851–1279] vs. 861 ng/mL [806–979], *p* < 10^−4^), sP-selectin levels did not differ (155 ng/mL [129–188] vs. 156 ng/mL [144–177], *p* = 0.48). None of the multivariable regression models was valid for the prediction of sVCAM-1 and sP-selectin in CF.

**Conclusions:**

We found higher sVCAM-1 concentrations in CF patients than in healthy subjects, which were not explained by CF characteristics. Further research is required to check whether sVCAM-1 is a marker of microangiopathy in CF.

## Introduction

The increasing survival of cystic fibrosis (CF) patients draws attention to CF-related cardiovascular risk. It may be expected that the CF-related chronic inflammation of the respiratory tract, diabetes, decreased antioxidant levels, altered fatty acid profile [1], gut dysbiosis, and dysregulated immune responses will promote atherogenesis. Recent studies demonstrated endothelial dysfunction and increased arterial stiffness are present in young cystic fibrosis patients [2, 3]. Findings from the murine model of CF point towards the existence of inherent causes of cardiovascular disease in CF, which would be independent of the bronchopulmonary disease [4]. Nevertheless, still little is known about CF-related risk of cardiovascular disease and its determinants.

Among proposed biomarkers of cardiovascular risk are cell adhesion molecules—soluble vascular cell adhesion molecule 1 (sVCAM-1; CD106) and soluble P-selectin (sP-selectin; CD62). VCAM-1 is crucial for leukocyte adhesion to the endothelium [5] and has a broad clinical relevance. In a group of 1246 patients with coronary artery disease followed for a mean of 2.7 years sVCAM-1 was independently associated with an increased risk of death from cardiovascular complications (2.8-fold in the upper vs. the lower quartile) and the identified effect could not be predicted by high-sensitivity C-reactive protein (hsCRP) [6]. In the Second Manifestations of ARTerial disease (SMART) study (*n* = 1002), sVCAM-1 was associated with the risk of cardiovascular events [7]. In the Bruneck study, which followed 880 persons over 20 years, sVCAM-1 predicted the occurrence of atrial fibrillation after adjusting for potential confounders [8]. Plasma sVCAM-1 was shown to correlate with higher resting cerebrovascular resistance and poorer cognitive function [9].

Higher concentrations of sVCAM-1 and sP-selectin are found in the plasma of patients with peripheral artery disease than in age-matched healthy subjects [10]. de Faria et al. showed that both plasma sVCAM-1 and sP-selectin are related to greater arterial stiffness and cardiac hypertrophy. In the same study, sP-selectin indicated target organ damage even after adjustment for blood pressure [11]. Serum sVCAM-1 as well as sP-selectin are higher in metabolic syndrome patients compared to age-matched persons without it; they also positively correlate with carotid intima-media thickness [12].

A study performed in 345 healthy women revealed that sP-selectin correlates with a greater risk of cardiovascular events (2.2-fold in the upper vs. the lower quintile). The observed effect is independent of the established cardiovascular risk factors [13]. In European Prospective Investigation into Cancer and Nutrition‐NL higher sP-selectin was linked to an increased risk of cardiovascular events (*n* = 288) [7]. In persons with left ventricular ejection fraction >50%, plasma sP-selectin was shown to predict cardiac events [14]. In men at higher risk of cardiovascular disease, sP-selectin associates with the presence of carotid plaque [15]. In non-Hispanic white Americans, over a 10-year follow-up, baseline plasma sP-selectin was related to the future risk of coronary heart disease. It was also associated with greater coronary artery calcium, glycated hemoglobin [16], and—in another study—with lower ankle-brachial index [17].

Despite microvasculature dysfunction in CF [18] the available data on soluble cell adhesion molecules in CF are scarce. De Rose et al. identified increased intercellular adhesion molecule 1 (ICAM-1; CD54) and E-selectin (CD62E) concentrations in CF patients [19]. However, in the same subjects, they found normal levels of sVCAM-1. An inverse correlation between forced expiratory volume in 1 s (FEV1) and sP-selectin was also described [20] as well as higher sP-selectin levels in CF compared with healthy controls [21]. The above findings come from few studies with moderate sample sizes. A comprehensive analysis of potential clinical correlates of soluble cell adhesion molecule concentrations in CF is also lacking.

We hypothesized that serum sVCAM-1 and sP-selectin concentrations differ between patients with CF and healthy subjects (HS). The secondary hypothesis was that the clinical characteristics of CF predict the levels of the above biomarkers. Thus, we aimed to fill the gaps in our knowledge of two molecular factors potentially linked to cardiovascular health in clinically stable CF.

## Methods

This study is a part of the AtheroCF project, which aims at understanding the background of atherosclerosis in CF. Patients were recruited in tertiary care centers in Poznan, Rabka, Gdansk, and Warsaw (Poland) between June 2013 and June 2016 [22]. The inclusion criteria comprised CF diagnosed according to CF Foundation Guidelines [23] and age ≥16 years. Exclusion criteria for CF patients and HS were common: a family history of hypercholesterolemia and/or hypertriglyceridemia and/or cardiovascular event before 65 (women) or 55 years of age (men).

Serum hsCRP concentration was determined with immunoturbidimetry (Cobas, Roche, Rotkreuz, Switzerland). Serum soluble VCAM-1 (sVCAM-1) and sP-selectin levels were assessed using ELISA (DRG Instruments GmbH, Marburg, Germany).

All CF patients were clinically stable i.e., did not have acute exacerbation of the bronchopulmonary disease. FEV1% values were taken from current patients’ clinical records (last 6 months). CFTR mutations were divided into severe (class I or II) and other. Exocrine pancreatic status was determined using fecal elastase-1 (ELISA; Schebo Biotech, Giessen, Germany) [24, 25]. CF-associated liver disease was diagnosed according to guidelines by Debray et al. [26]. Diabetes was diagnosed by physicians specialized in diabetes care. *Pseudomonas aeruginosa* status was considered positive if chronic or recurrent infections were confirmed by culture [27].

The study was conceived to detect a difference equaling ½ of standard deviation, assuming the 0.80 power of the test and the significance level set at 0.05 (target sample size 100 vs. 50 cases). Statistical analyses were carried out in Statistica 12 (StatSoft Inc., Tulsa, USA). The Shapiro–Wilk test was used to check the data for normality of the distribution and the F-test to verify whether variances were equal (not shown). Medians [1st–3rd quartiles] are reported. The Mann–Whitney *U*-test was employed to compare parameter values between groups. Spearman’s rank-order correlation was calculated. Multivariable linear regression models were built in order to adjust for confounding and identify potential independent correlates of the two biomarkers (all effects). The first set of regression models included all the measured parameters with the exception of diabetes mellitus (all diabetic patients had exocrine pancreatic insufficiency); the second set investigated the following selected parameters: age, sex, FEV1%, BMI, severe mutation, and exocrine pancreatic insufficiency.

All volunteers gave their informed written consent to participate in the study; in the case of adolescents, the informed written consent was also given by patients’ parents. The study respected the revised Declaration of Helsinki and was approved by the Bioethical Committee at Poznan University of Medical Sciences (decision no. 250/10). The structure of this article is based on the Strengthening the Reporting of Observational studies in Epidemiology (STROBE) checklist [28].

## Results

One hundred-eight patients with CF and 51 HS were recruited for the study. The genotypes are listed in Table 1; 56 of these (52%) contained one or two class I or II mutations and were considered to predispose to severe course of the disease. Median FEV1% [1st–3rd quartile] was 61% [46–84%]. Eighty-eight patients (81%) had exocrine pancreatic insufficiency, of them, 24 had diabetes mellitus (22%), 42 had CF-related liver disease (39%), and 67 had positive *P. aeruginosa* status (62%). The only missing data were two FEV1% measurements; we did not include these cases in regression analyses (listwise deletion).

**Table 1:** Cystic fibrosis transmembrane conductance regulator (*CFTR*) genotypes of the 108 cystic fibrosis patients enrolled for the study

*n*	Genotype
32	F508del/F508del
15	Unknown/unknown
11	F508del/unknown
8	F508del/3849+10kbC->T
4	F508del/CFTRdele2,3
3	F508del/3272-26A->G
2	F508del/1717-1G->A, F508del/W1282X
1	F508del/2143delT, F508del/2183AA->G, F508del/2721del11, F508del/3121-2A->G, F508del/3171insC, F508del/3600+2insT, F508del/G551D, F508del/N1303K, F508del/R117H, F508del/R352Q, F508del/R553X, F508del/R851X, CFTRdele2,3/CFTRdele2,3, CFTRdele2,3/3849+10kbC->T, CFTRdele2,3/unknown, CFTRdele2,3/N1303K, N1303K/G551D, N1303K/unknown, 1524+1G->A/3944delGT;406-6T->C, 1717-1G->A/unknown, 2183AA-G/1717-1G->A, 2184insA/unknown, 3272-26A>G/unknown, 3659delC/R153I, 3849+10kbC->T/3600+1G>T, S1196X/Q138X, T581l/2721del11

**Fig. 1:**
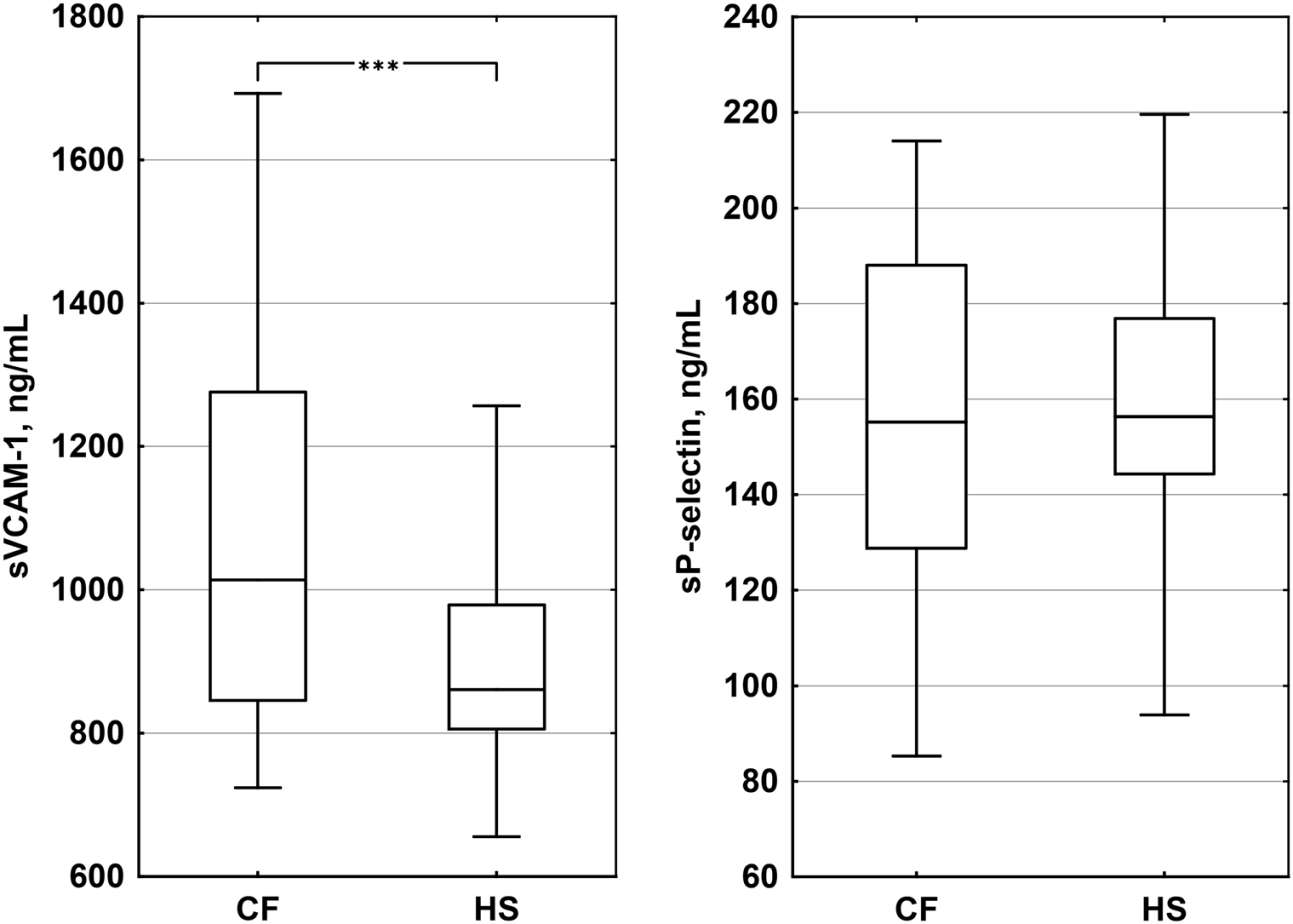
Boxplots illustrating the concentrations of soluble vascular cell adhesion molecule 1 (sVCAM-1) and soluble P-selectin (sP-selectin) in cystic fibrosis (CF) patients and healthy subjects (HS). Median values, 1st–3rd quartiles, and 5th–95th percentiles are shown. *Asterisks* indicate statistical significance (***p < 10^−4^)

Basic characteristics and biomarker values in CF and HS are compared in Table 1. In CF higher hsCRP and sVCAM-1 concentrations were found. sVCAM-1 and sP-selectin levels in CF and HS are compared in Fig. 1.

Extreme outliers that could disproportionately influence the regression models were excluded after residual analysis (one sVCAM-1 and one sP-selectin concentration). There were no significant collinearity issues. None of the models for the prediction of the two investigated biomarkers was valid. No correlations were found between FEV1% or hsCRP and the two biomarkers. They did not correlate with each other either. In an explorative analysis, sVCAM-1 concentration was higher in CF patients with exocrine pancreatic insufficiency compared with those who were pancreatic-sufficient (1043 ng/mL [897–1306] vs. 895 ng/mL [781–1007], *p* = 0.003); among pancreatic-insufficient patients there were no differences between diabetics and non-diabetics (1151 ng/mL [916–1327] vs. 1026 ng/mL [899–1304], *p* = 0.48) (Table 2).

**Table 2:** Group characteristics and comparison of soluble vascular cell adhesion molecule 1 (sVCAM-1) and soluble P-selectin (sP-selectin) in cystic fibrosis (CF) patients and healthy subjects (HS)

Parameter	CF, *n* = 108	HS, *n* = 51	*P*
Sex (F/M)	61/47	31/20	0.73^a^
Age (years)	22.0 (19.1–31.0)	24.1 (21.7–28.1)	0.38
Body mass index (kg/m^2^)	20.5 (18.4–22.2)	21.6 (19.9–23.4)	0.02
hsCRP (mg/L)	3.6 (1.1–7.1)	0.5 (0.3–1.0)	<10^−10^
sVCAM-1 (ng/mL)	1018 (851–1279)	861 (806–979)	<10^−4^
sP-selectin (ng/mL)	155 (129–188)	156 (144–177)	0.48

Median values [1st–3rd quartiles] are presented

*F* female, *hsCRP* high-specificity C-reactive protein, *M* male

^a^Fisher’s test, two-tailed *p* value

## Discussion

This study provides new data on soluble cell adhesion molecules in cystic fibrosis. While sVCAM-1 concentration was higher in CF than in HS, and that of sP-selectin did not differ significantly between the two groups.

### sVCAM-1

VCAM-1 is a ligand of very late antigen-4 (CD49d/CD29; integrin α4β1), taking part in adhesion of leukocytes to the endothelium [29]. It was found to associate with coronary artery disease [30] and its concentrations were shown to be higher in patients with hypertension [31], obesity, and diabetes [32], and also in women with preeclampsia [33]. sVCAM-1 levels were also raised in diabetic women in early pregnancy having both retino- and nephropathy compared with those who did not have retinopathy [34]. Retinopathy associated with high sVCAM-1 concentrations in another study as well, suggesting a link not only to microangiopathy through endothelial damage, but also to neovascularization [35].

In renal-insufficient patients without diabetes and atherosclerosis, sVCAM-1 was correlated with carotid intima-media thickness [36]. In menopausal women, blood-borne microvesicle VCAM-1 was weakly associated with a positive change in carotid intima-media thickness over a period 4 years; microvesicle P-selectin was included in the principal component, predicting increases in reactive hyperemia [37]. A 6-month follow-up of 75 acute coronary syndrome patients demonstrated that sVCAM-1 levels predict the risk of future major cardiac events (OR 4.62; 95% CI 1.8–11.4) [38]. High serum sVCAM-1 associates with coronary artery disease as well as lower brain-derived neurotrophic factor values during oral glucose tolerance test [39]. VCAM-1 relates to the thickness of the carotid intima-media and its plaque in rheumatoid arthritis [40]. In women, serum sVCAM-1 is inversely correlated with visceral adipose tissue [41]. In persons with hyperglycemia, moderate exercise was shown to increase sVCAM-1 [42].

In a murine model of reduced VCAM-1 expression, the burden of aortic lesions was 48% lower compared with a control group [72]. In estrogen-deficient rats, sVCAM-1 decreased after augmenting the content of polyunsaturated fatty acids in the diet; this was also accompanied by reductions in both leukocyte adherence to the wall of the aorta and platelet adhesiveness [43]. VCAM-1 is overexpressed in atherotic plaque after acute hypoxia and its silencing with interfering RNA in vivo decreases granulocyte recruitment to the damaged tissue [44]. Interestingly, the association between a plasma-specific microRNA—miR-1185—and arterial stiffness was found to be partially mediated by VCAM-1 [45].

It should, however, be admitted that there is important evidence contrary to sVCAM-1 being an independent risk factor for cardiovascular events [46, 47]. Both soluble and membrane VCAM-1 are unspecific. VCAM-1 expression is raised in acute respiratory distress syndrome [48], in breast [49] and non-small cell lung cancer [50], and in rheumatoid arthritis [51], where it decreases following treatment with infliximab and methotrexate [52]. Plasma sVCAM-1 is known to respond to the exposure to air pollution with fine particulate matter (PM_2.5_) [53]. The available research on the role of this molecule is mainly clinical. Overall, in patients with substantial endothelial damage sVCAM-1 may be considered a risk factor of cardiovascular disease and in other subjects an indicator of atherosclerosis progression [54].

We did not confirm the findings by De Rose et al., who found that sVCAM-1 levels did not differ in 29 patients with CF and 12 healthy volunteers [19]. In the light of the above-mentioned studies, it might be proposed that the higher sVCAM-1 concentration in CF—as found in this study—may reflect a state of chronic inflammation, which probably predisposes to cardiovascular disease.

sVCAM-1 concentrations found by various studies differ and may not be comparable. For instance, its serum levels in HS in our study were similar to that found by some other research groups [43, 55, 56]. They are, however, higher than reported in a number of other publications [30, 33, 57]. We suppose that this variability might be due to specificity of antibodies used in ELISA kits. In fact, while the main sVCAM-1 form has the molecular weight of about 100 kDa, smaller forms also exist. Curiously, Hahne et al. showed that in mice it was 42-kDa sVCAM-1 and not 100-kDa sVCAM-1 that responded to stimulation with lipopolysaccharide [58]. Garton et al. indicated that it is the 100-kDa sVCAM-1 that is predominant in the serum of mice and that it was cleaved from the cellular surface by ADAM (a disintegrin and metalloproteinase) metallopeptidase domain 17 (ADAM17) in response to stimulation by 12-*O*-tetradecanoylphorbol-13-acetate [59]. They also revealed that neutrophil elastase produces a 65-kDa sVCAM-1 cleavage product. Singh et al. demonstrated that ADAM17-mediated sVCAM-1 shedding could be cytokine-induced [60], but did not relate to the molecular weight of obtained sVCAM-1. While some antibody suppliers state that their antibodies yield only the 100 kDa band, we also found others that admitted that the antibodies detected unidentified 48–49-kDa proteins. Therefore it cannot be excluded that there is a systematic bias introduced by various ELISA kits, some of which might recognize only the epitopes that are present in large, but not in smaller forms of sVCAM-1. This renders difficult the direct comparisons of sVCAM-1 concentrations obtained in different studies.

### sP-selectin

P-selectin enables leukocyte adhesion to the vascular wall [61]. Its major ligands include P-selectin glycoprotein ligand-1 and TIM-1—T cell immunoglobulin and mucin domain 1 [62, 63]. The role of sP-selectin in atherosclerosis development and its association with the risk of venous thromboembolism is supported by studies of *SELP* polymorphisms [64, 65] and research in animal models [66]. Additionally, *SELP* rs6128 major allele is associated with a higher sP-selectin concentration and diabetic retinopathy [67]. In dialysis patients, raised sP-selectin concentrations associate with atherosclerotic cardiovascular disease and mortality [68]. The Framingham Heart Study Offspring and Omni studies revealed correlates of sP-selectin levels: male sex, age, cigarette smoking, and other modifiable cardiovascular risk factors [69]. sP-selectin is also more abundant in the blood (serum and plasma) of patients with severe chronic venous insufficiency [70]. In community-acquired pneumonia plasma sP-selectin is increased [71] and predicts the occurrence of myocardial infarction [72]. Platelet P-selectin is associated with hypertension [73]; in patients with hypertension caused by primary aldosteronism serum, sP-selectin decreased after removal of aldosterone-secreting adrenal adenoma [74]. Plasma sP-selectin associates with waist-to-hip ratio and visceral adipose tissue in men [41]. In healthy persons, physical effort relates to lower sP-selectin [75]. However, it must be underscored that there are also large cohort studies, in which plasma sP-selectin was not linked to cardiological outcomes [7, 76].

P-selectin interaction with its ligand is considered a target for novel therapies aiming at the reduction of cardiovascular risk [77]. Inclacumab, which is a monoclonal antibody against P-selectin, protects against myocardial damage after reperfusion in non-ST-segment elevation myocardial infarction [78]. Platelet P-selectin expression is reduced by thienopyridine class inhibitors of the adenosine pyrophosphate receptor [79]. This is relevant because platelet-derived microparticles reduce the plasticity of FOXP3(+) regulatory T cells by interacting with their P-selectin(+) subset, creating a pro-inflammatory milieu [80].

Serum sP-selectin levels tend to be higher than in plasma since they include P-selectin released from platelets activated during clot formation. The correlation between serum and plasma sP-selectin concentrations was shown to be linear and moderate-to-strong. Moreover, the reproducibility of measurements in both serum and plasma is excellent (intraclass correlation coefficient 0.98 and 0.92, respectively). Serum and plasma sP-selectin does not correlate with platelet count or mean platelet volume [81].

It seems that the role of P-selectin is dependent on where it is localized: in the serum, the platelets, or the endothelium. A cross-sectional study by Cleanthis et al. indicated that soluble—but not platelet—P-selectin correlated with spontaneous platelet aggregation in patients with intermittent claudication or stroke [82]. On the other hand, platelet—and not endothelial—P-selectin is required for the development of acute lung injury after a chemical insult: it mediates a platelet-neutrophil interaction leading to thromboxane A2 production, neutrophil adhesion, and—consequently—greater tissue damage [83].

O’Sullivan et al. found that P-selectin expression on platelets obtained in CF patients was insignificantly greater before stimulation with adenosine diphosphate, significantly higher after this stimulation at all five concentrations employed, and not entirely abolished by prostaglandin E1, which was the case in normal platelets [84]. The incomplete inhibition of platelet aggregation in CF was known previously [85]. Because similar findings were reproduced in washed platelets, O’Sullivan et al. concluded that this was due to an intrinsic platelet property. Curiously, O’Sullivan et al. did not identify CFTR or its mRNA within normal platelets, which led them to propose that the observed CF platelet hyperactivity could be traced back to megakaryocyte or related to a different chloride channel.

Sturm et al. found a higher sP-selectin concentration in 54 CF patients—including children—compared with 55 age- and sex-matched healthy subjects [21]. In the subgroup of subjects aged 15–41 years the values compared as follows (*n*_CF_ = 28; *n*_HS_ = 29): 41 ng/mL [25–56] versus 29 ng/mL [17–60]. A similar difference was observed in children aged 3–14 years. Sturm et al. considered sP-selectin to be a platelet-derived inflammatory factor. Another study, by Romano et al., reported sP-selectin to be higher in 20 CF patients compared with 20 HS [20] and to correlate with worse FEV1%. In our study, sP-selectin levels in patients and HS did not differ and the medians were within the reference range established by Deneva-Koycheva et al. (102–210 ng/mL in the serum) [57].

Whereas Sturm et al. assessed the level of sP-selectin in the plasma, we measured serum P-selectin: initially present in the plasma and released from platelets on clot formation. This partly explains the higher values in our study and may hint at why Sturm et al. and Romano et al. found an effect while we did not. However, the difference between serum and plasma sP-selectin in a study by Valdes et al. was not threefold as in this case but twofold [81]. Considering that median CRP levels and FEV1% in the group assessed by Sturm et al. and in ours were very similar, and that they also included only clinically stable patients, we expect that our assessment methods may have differed as well.

An unsurprising finding is the increased hsCRP concentration. Although the CF patients were clinically stable, it seems plausible that their hsCRP levels reflect a chronic inflammatory process in the respiratory tract. This process may be expected to negatively affect cardiac health.

The main strengths of this research include a relatively large sample size and a comprehensive characterization of CF-related clinical factors. Its main limitations comprise: the cross-sectional design, which does not permit for establishment of causation, and the investigation of biomarkers, which may give insight into pathophysiology, but cannot replace clinical endpoints. It must also be considered that the varying sensitivity of the available tests (various ELISA kits and other antibody-based techniques) lowers the utility of direct comparisons of soluble cell adhesion molecules’ concentrations between studies.

## Conclusions

We found higher sVCAM-1 concentrations in CF patients than in HS, which could not be explained by CF characteristics. Further research is required to check whether sVCAM-1 is a marker of microangiopathy in CF.
